# Photosensitizing Furocoumarins: Content in Plant Matrices and Kinetics of Supercritical Carbon Dioxide Extraction

**DOI:** 10.3390/molecules25173805

**Published:** 2020-08-21

**Authors:** Łukasz Woźniak, Marzena Połaska, Krystian Marszałek, Sylwia Skąpska

**Affiliations:** 1 Department of Fruit and Vegetable Product Technology, Institute of Agricultural and Food Biotechnology, 36 Rakowiecka Street, 02532 Warsaw, Poland; krystian.marszalek@ibprs.pl (K.M.); sylwia.skapska@ibprs.pl (S.S.); 2 Department of Microbiology, Institute of Agricultural and Food Biotechnology, 36 Rakowiecka Street, 02532 Warsaw, Poland; marzena.polaska@ibprs.pl

**Keywords:** *Angelica archangelica*, broken plus intact cell model, *Citrus paradisi*, *Cnidium monnieri*, green chemistry, *Psoralea corylifolia*, supercritical fluid extraction

## Abstract

Furocoumarins are a group of plant phytoalexins exhibiting various bioactive properties; the most important of which are photosensitization and alteration of P450 cytochrome activity. Supercritical fluid extraction with carbon dioxide has been proposed as a green alternative for an organic solvent extraction of the furocoumarins. Four plant matrices rich in furocoumarins were extracted with CO_2_ at a temperature of 80 °C and pressure of 40 MPa, as these conditions were characterized by the highest solubility of furocoumarins. The extracts collected were analyzed using the HPLC method and the results obtained were used for the mathematical modeling of the observed phenomena. The total content of the furocoumarins in the matrices was 4.03–26.45 mg g^−1^ of dry weight. The impact of the process parameters on the solubility was consistent with the Chrastil equation. The broken plus intact cell model proved to be suitable to describe extraction curves obtained. The research proved the possibility of supercritical carbon dioxide utilization for the extraction of the furocoumarins from plant material and provided valuable data for prospective industrial-scale experiments.

## 1. Introduction

Furocoumarins (also known as furanocoumarins) are a group of plant secondary metabolites exhibiting several pharmacological activities. The largest quantities of these compounds are found in plants of the Rutaceae and Apiaceae families. Until now, more than one thousand furocoumarins of natural origin have been described, while a few hundred more have been obtained by chemical synthesis [1]. The furocoumarins act as phytoalexins—a biochemical defense system triggered by stress factors, such as tissue damage or the infection of bacterial/fungal pathogens [2,3,4].

The most characteristic bioactive feature of furocoumarins—photosensitization—is based on their ability to react with nucleic acids. The combination of their planar aromatic structure and hydrophobic character promotes the intercalation of the DNA double helix. Subsequent excitation by ultraviolet radiation leads to the formation of an array of stable adducts between furocoumarins and nucleobases. The results of such reactions are necrosis, apoptosis, or hindering of cell replication, depending on the dose of furocoumarins and intensity of the radiation [5]. Therefore, the absorption of furocoumarins, e.g., via skin exposure or as a food constituent, could lead to severe sunburn [6,7]. On the other hand, the photosensitizing properties of furocoumarins are used in the therapy of several skin disorders. Since its introduction in the 1970s, photochemotherapy has become one of the primary methods for the treatment of psoriasis, eczema, and vitiligo [5,8], even after concerns of the increased risk of skin cancer were raised [9,10]. The literature provides information on the numerous biological activities attributed to furocoumarins, including fungicidal, antiviral, anticancer, and analgesic properties [1,11]. Certain furocoumarins are also capable of altering the activity of the P450 cytochrome enzyme; the resulting changes in drug metabolism are commonly called the “grapefruit juice effect” [12]. Taking into consideration the above-mentioned activities of furocoumarins, it can be stated that their presence may be beneficial in certain circumstances and undesirable in others. Therefore, the extraction of furocoumarins from plant material may be part of the isolation of bioactive molecules or the removal of potentially harmful food constituents.

Supercritical fluid extraction (SFE) is a green method of separation that has gained remarkable interest among the scientific community in recent years. SFE can be more advantageous than traditional extraction methods due to the higher mass transfer rates and increased selectivity. Carbon dioxide is the solvent of choice in the majority of the applications because of its beneficial features: temperate critical point parameters, minimal toxicity, chemical inertness, and low cost. Several hundred plant matrices have already been subjected to extraction using supercritical carbon dioxide, although the number of industrial applications of SFE is still limited [13,14]. A few research teams have already investigated the concept of SFE of furocoumarins from plant materials; however, most of the authors only focused on obtaining of the extracts, their characterization, and the selection of process parameters, whereas they did not investigate the kinetics of the process, which is essential for practical implementation of the process on an industrial scale. The research conducted by Wang et al. [15] focused on optimizing the extraction and purification of psoralen and isopsoralen from *Psoralea corylifolia* seeds. The best results were obtained at a pressure of 26 MPa and temperature of 60 °C. The authors conducted a larger scale experiment using optimized conditions; however, the extraction kinetics was not investigated. A few other publications used SFE as a sample preparation step during a phytochemical analysis of the samples; the optimized conditions reported in these papers were in the range of 20–38.5 MPa and 40–70 °C [16,17,18,19]. The use of supercritical carbon dioxide for desorption of citrus peel oils was investigated by a group of French and Italian researchers. Their goal was to increase the quality of the oils by the selective elimination of unwanted compounds (including furocoumarins). Similar extraction parameters were used for all the oils tested: temperature of 40 °C and pressure in the range of 7.5–10 MPa. Under these conditions, carbon dioxide was unable to dissolve the furocoumarins, while the majority of terpenes responsible for aroma were extracted, resulting in purified oil being obtained [20,21,22].

According to the authors’ best knowledge, this paper is the first that comprehensively reports on the kinetics of the furocoumarin extraction from plant resources. The experiments included an evaluation of the furocoumarin content in selected matrices, an estimation of the solubility of analytes, and a modeling of the process. The results obtained may become the starting point for the industrial implementation of the process.

## 2. Results and Discussion

### 2.1. Furocoumarin Content in Plant Matrices

**Figure 1:**
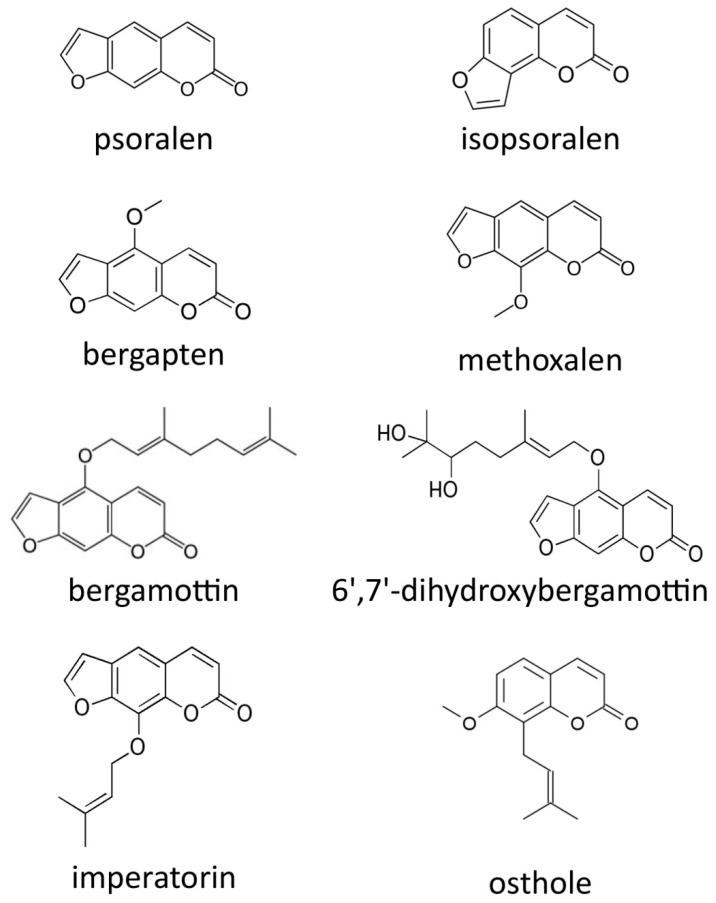
Furocoumarins identified and quantified in analyzed plants.

The first task was the qualitative and quantitative characterization of furocoumarins present in selected matrices. Soxhlet extracts were obtained and analyzed using the HPLC method; compounds were identified by comparing their retention time with analytical standards. The structures of the identified analytes are shown in Figure 1, while the furocoumarin content is presented in Table 1.

The unidentified compounds were classified as furocoumarins based on spectrofluorometric data obtained during the HPLC analysis (strong fluorescence at an excitation wavelength of 310 nm and an emission range of 440–540 nm). The literature provides data on minor constituents of the analyzed plant material and several more reference standards of the furocoumarins are available through commercial sales; however, the analysis was limited to the most abundant furocoumarins as this research is focused on the extraction kinetics. Furthermore, the unidentified compounds accounted for less than 10% of the total furocoumarin content.

*Psoralea corylifolia* was the most abundant source of furocoumarins with a total content of over 2.5% of mass; the two main compounds were identified as psoralen and isopsoralen. The composition pattern observed, with two dominant compounds, is reported in the majority of relevant literature data [15,23]. According to data in the literature, minor constituents of *P. corylifolia* include, inter alia, bakuchicin, and psoralidin [23]. Some reports state that methoxalen can also be found [24]; however this compound was not identified in our study.

The seeds of *Cnidium monnieri* and the root of *Angelica archangelica* had similar total furocoumarin content and identified compounds; however, the quantitative patterns differed. Osthole was the most abundant furocoumarin in *C. monnieri* similarly to data in the literature; the reported minor constituents included, among others, isopimpinellin, auraptenol, and morinzin [25,26]. Imperatorin was identified as the primary furocoumarin of *A. archangelica* in the literature; unidentified compounds might include pimpinellin and phellopterin [27,28,29].

The experiments showed that grapefruit pomace contained the least furocoumarins of the matrices analyzed; DHB was the most prevalent compound followed by bergamottin and bergapten. Similar results were reported in all fruits from the *Citrus* genus [30,31,32]. The unidentified compounds presumably include bergaptol and epoxybergamottin, which are intermediates of the more abundant furocoumarins biosynthesis [31].

### 2.2. Selection of Extraction Parameters

Nine combinations of temperature (40, 60, and 80 °C) and pressure (10, 20, and 40 MPa) were selected for the experiments. Determination of the solubility was performed in duplicate using the method presented in Section 3.3.3. The total content of extracted furocoumarins as well as the content of individual compounds were analyzed and used to select the optimal process parameters.

All furocoumarins exhibited the highest solubility in supercritical carbon dioxide at a temperature of 80 °C and pressure of 40 MPa. The solubility, under these conditions, of the main furocoumarins (occurring in the matrices at a level of 0.5 mg g^−1^ or higher) is listed in Table 2. The results obtained show a relationship between the structure of the analyte and its solubility. Psoralen and isopsoralen were characterized by similar solubility; a bigger impact was observed for side chains altering the polarity of the molecules. The presence of the methoxy group in methoxsalen and bergapten lowered their solubility by approximately 30%, while prenyloxy and geranyloxy groups in imperatorin and bergamottin increased their solubility by 35% and 90%, respectively. The oxidation of bergamottin to DHB resulting in the presence of two hydroxy groups led to a more than six-fold decrease in solubility. The reported solubility values were impacted by other matrix constituents and therefore they probably vary from ones that would be obtained in furocoumarin-carbon dioxide binary systems. The impact of each matrix on the results observed was different as their composition varies and therefore observed solubilities of methoxsalen and imperatorin differs between *C. monnieri* and *A. archangelica*. The sum of individual furocoumarin solubilities varied between the matrices.

**Figure 2:**
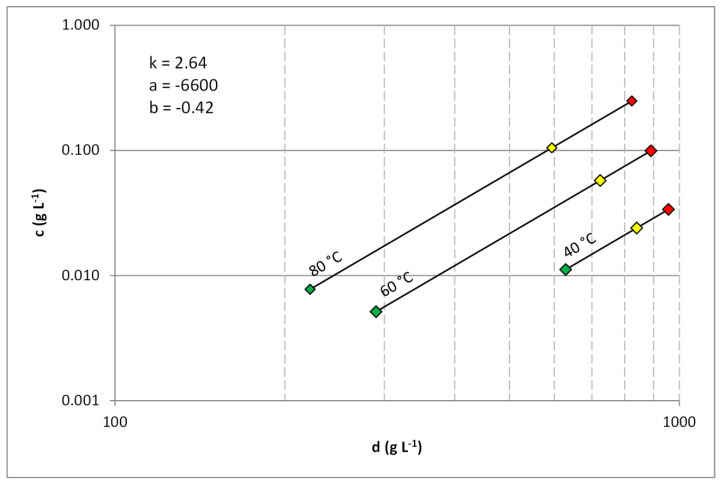
Impact of temperature of supercritical carbon dioxide and its density (*d*) on the solubility (*c*) of psoralen. The colors of the points represent the pressure used: green—10 MPa, yellow—20 MPa, red—40 MPa.

The impact of the process parameters on the solubility of analytes was evaluated using an equation proposed by Chrastil (Equation (1)) [33]. The equation connects the solubility of an analyte with temperature and density of a supercritical fluid in the simple binary system. However, as reported in one of our earlier publications [34], the equation may be suitable to describe the complex phenomena occurring during solubilization of constituents of complex matrices. The measurements conducted using plant matrices instead of pure analytes allowed us to include the impact of other constituents of analyzed plants that can impact solubility of furocoumarins. Figure 2 presents the linear relationships between carbon dioxide density and the solubility of psoralen in a log–log plot.

The relationship between the solubility and process parameters was similar in all the matrices, although the suitability of the Chrastil equation to describe the data obtained varied between the analytes. The equation accurately fits the data for unbranched (psoralen and isopsoralen) and methoxy substituted (bergapten and methoxsalen) furocoumarins; the average relative difference between the data and model was in the range of 2.4–5.3%. On the other hand, for prenyloxy and geranyloxy substituted compounds (bergamottin, imperatorin, and osthole) the average relative difference was in the range of 19.5–26.3%, indicating that in this case behavior of the solvent–solute is strongly affected by other matrix constituents limiting the usability of the Chrastil equation. An evaluation for DHB was not possible since the amount extracted for six sets of the examined parameters was too low for quantification even if the amount of plant material was increased. Knowledge on the DHB solubility would be interesting from the scientific point-of-view; although, from a practical point-of-view the solubility at these conditions is too low to be effectively used in an industrial implementation of the process.

### 2.3. Extraction Kinetics

**Figure 3:**
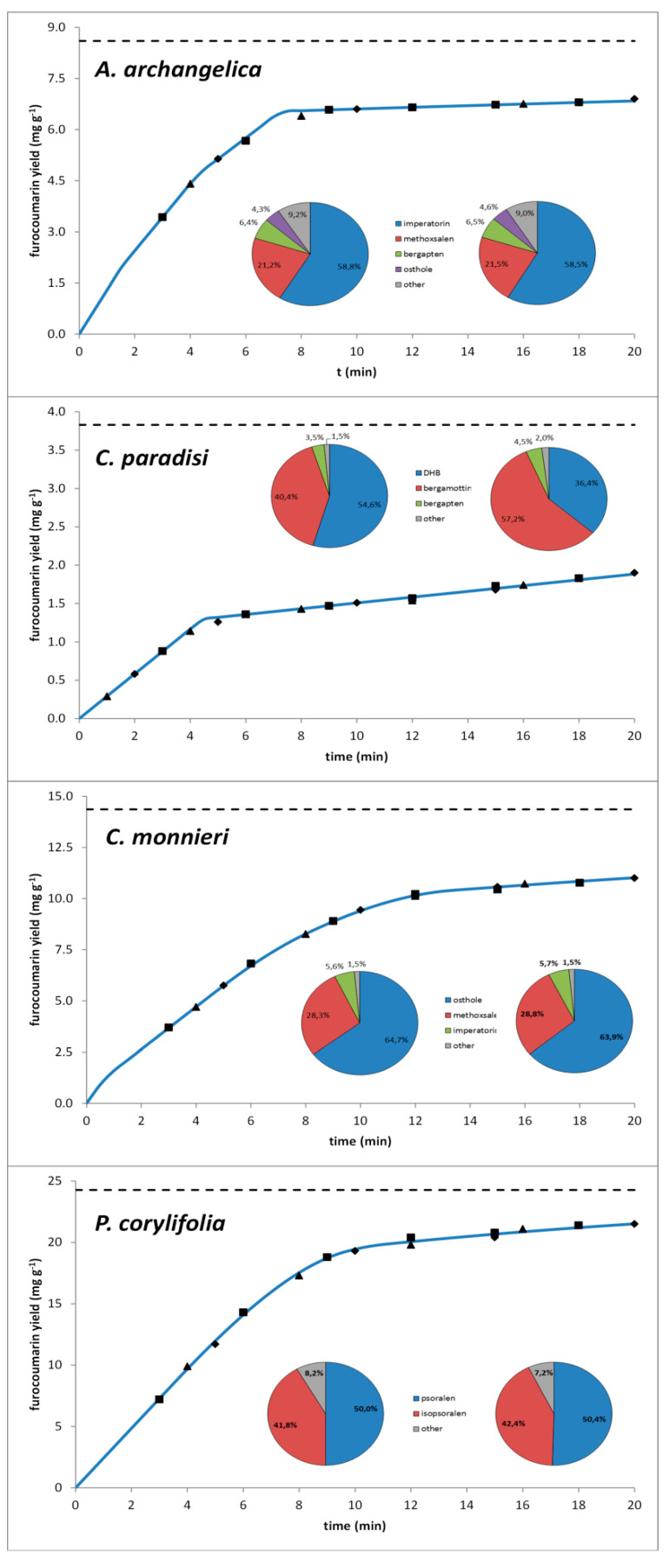
Extraction kinetics of furocoumarins from plant tissues at a temperature of 80 °C and pressure of 40 MPa. The blue lines represent the yield predicted using the broken plus intact cell model, the black points (squares, triangles, and diamonds)—experimental data from three processes, the black lines content of analytes in samples, pie charts—furocoumarin profiles in raw materials (left) and extracts obtained (right).

The supercritical extractions were conducted at a temperature of 80 °C and pressure of 40 MPa because, under these conditions, supercritical carbon dioxide showed the highest solubility of all compounds of interest. The broken plus intact cell model was used to analyze the extraction kinetics; the parameters obtained during the calculations are listed in Table 3. Figure 3 shows a comparison of the extraction curves obtained during modeling with experimental data; it also presents a comparison between the furocoumarin profiles in the matrices and in extracts obtained using supercritical carbon dioxide. Additional information on analyzed matrices and bed characteristics are listed in Table S1 in Supplementary Materials.

The broken plus intact cell model was an appropriate method to describe the extraction kinetics. The average relative difference between empirical data and model values was in the range of 3.1–6.2%. The values of the kinetic parameters for furocoumarins were not reported in the literature yet; however a comprehensive review paper by Huang et al. presents data obtained for other groups of compounds. Obtained values of *k_f_* and *k_s_* have the same order of magnitude as the majority of literature findings, however lone reports showing values approximately 100-fold higher or lower can also be found; literature values of *r* parameter are in a very wide range from close to zero (0.020) to close to one (0.992) [35]. Unbranched compounds were characterized by the highest mass transfer ratios, while furocoumarins from *C. paradisi* with large geranyloxy substituents were characterized by the lowest ratios.

The qualitative profiles of the furocoumarins in the extracts obtained were similar to the raw plant material; varying profiles were observed only in the case of *C. paradisi* due to differences in the solubility of DHB and bergamottin. The differences in the extraction kinetics between the matrices can be explained by the variation in the initial content of the furocoumarins, their solubility, the mass transfer ratios, and characteristics of the matrices (such as a specific surface area and a fraction of less accessible solute). Nevertheless, supercritical carbon dioxide at a temperature of 80 °C and pressure of 40 MPa was suitable as a solvent for extracting furocoumarins from the plant material.

Apart from furocoumarins the extracts contained other lipophilic groups of compounds present in the matrices. Due to expected diversity of extracted compounds only the total mass of the isolated fraction was analyzed. Typical classes of compounds that are extracted by supercritical carbon dioxide include: triglycerides, terpenoids, sterols, and fat-soluble vitamins [13]. Obtained data is presented in Figure S1 in Supplementary Materials. Furocoumarins constitute a minor fraction of obtained extracts; their content in Soxhlet extracts was in a range of 11–32% of the total extract obtained. The relative content of furocoumarins in supercritical carbon dioxide extracts was changing during the extraction as extraction rate varies between components of matrices. Nevertheless, the composition of extracts obtained with both methods differs significantly as supercritical carbon dioxide is much less polar than methanol. Fraction of furocoumarins in SFE extracts was in a range of 4.1–22.6%. The initial parts of obtained total extraction curves are very steep, which suggests that lipophilic compounds present in matrices are easily extracted by CO_2_.

**Figure 4:**
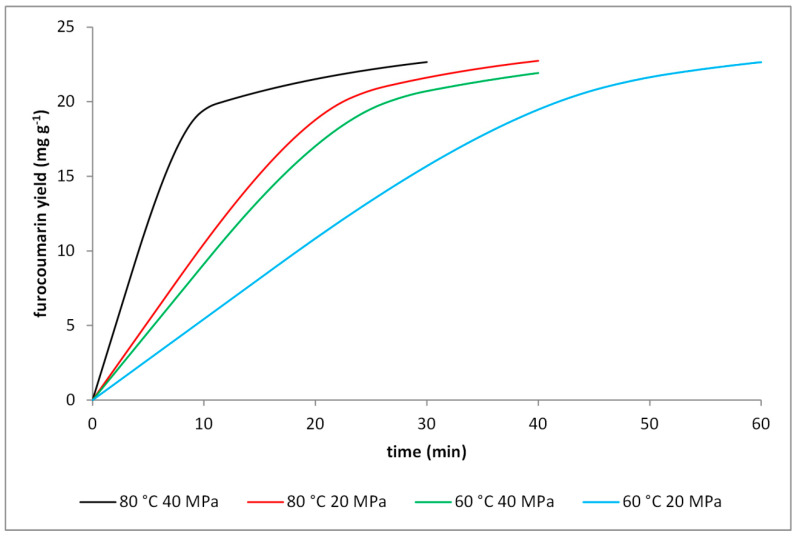
Extraction kinetics of furocoumarins from seeds of *P. corylifolia* at various temperatures and pressures. The lines represent the yield predicted using the broken plus intact cell model.

Highest solubility of analytes can be a decisive factor in the selection of the extraction parameters; however a limiting impact of mass transfer on the process cannot be ignored. Seeds of *P. corylifolia* were extracted using carbon dioxide under conditions characterized by lower solubility of furocoumarins in order to get deeper insight on process limitations. Obtained data was analyzed using a broken and intact cell model; the kinetic parameters obtained from calculations are shown in Table 4, while Figure 4 presents comparison of extraction curves for different carbon dioxide parameters. Mass transfer coefficients were affected by parameters of CO_2_. Two tendencies were observed: increase of coefficients with temperature, which can be attributed to a higher average speed of molecules in fluid; a decrease of coefficients at higher pressures can be connected with increased viscosity of carbon dioxide [36]. The curves obtained are characterized by a similar shape regardless of process parameters. The extraction of easily accessible solute is mainly limited by the solubility of furocoumarins; the latter stage of extraction is much slower and limited by mass transfer of analytes from intact cells. Extraction at 80 °C and 40 MPa proved to be superior to other tested parameter sets. Total extraction yield for abovementioned processes was also investigated. Obtained data is presented in Figure S1 in Supplementary Materials.

## 3. Materials and Methods

### 3.1. Materials

Four plant species, known for their high content of furocoumarins were chosen for experiments; their brief description is presented in Table 5. Grapefruit pomace was produced from fresh fruits and dried on a laboratory scale, while other products were acquired from local sellers.

Analytical grade: psoralen, isopsoralen, methoxsalen, bergapten, osthole, and imperatorin were acquired from Chromadex (Irvine, CA, USA), 6′,7′-dihydroxybergamottin (DHB) came from Toronto Research Chemicals (North York, ON, Canada), and bergamottin was from Sigma-Aldrich (Saint Louis, MO, USA). HPLC-grade methanol, acetonitrile, and tetrahydrofuran (THF) were bought from POCh (Gliwice, Poland). Technical grade carbon dioxide was acquired from a local supplier (Multax, Zielonki-Parcela, Poland).

### 3.2. Instrumentation

High-performance liquid chromatography analyses were conducted using equipment manufactured by Waters (Milford, MA, USA): 2695 Separation Module, 2996 Photodiode Array Detector, and 2475 Multi-Wavelength Fluorescence Detector. Supercritical carbon dioxide extractions were performed using SFE Spe-ed 4 apparatus (Applied Separations, Allentown, PA, USA). The plant material was ground using a laboratory mill (Predom, Niewiadów, Poland), the average diameter of the particles obtained was approximated using Olympus CX40 phase-contrast microscope (Olympus, Tokyo, Japan) and a Thoma chamber. The moisture content in samples was evaluated using HR83 Halogen Moisture Analyzer (Mettler Toledo, Columbus, OH, USA).

### 3.3. Extraction Procedures

#### 3.3.1. Supercritical Fluid Extraction (SFE)

Portions of milled plant material (approximately 10 g) were placed inside 24-mL extraction vessels and thermostated for thirty minutes prior to extraction. Next, the sample was percolated with a constant flow of CO_2_ using preselected values of temperature, pressure, and flow rate. Fractions of extracts were collected in beakers, which were changed in regular time intervals (once every 3, 4, or 5 min).

#### 3.3.2. Soxhlet Extraction

Soxhlet apparatus was used to determine the total furocoumarin content in the plant matrices. Milled samples (3–5 g) were extracted for 8 h with 250 mL of methanol, which ensured exhaustive extraction of furocoumarins from the plant material.

An additional experiment was performed to verify whether prolonged heating during Soxhlet extraction leads to thermal degradation of the furocoumarins. The plant samples were macerated with cold methanol and the extracts obtained were thermostated at 65 °C for 8 h. All the tested compounds were stable during heating.

#### 3.3.3. Determination of Furocoumarin Solubility in Supercritical Carbon Dioxide

The solubility of the selected furocoumarins in carbon dioxide at various pressure and temperature values was determined experimentally. Nine sets of parameters were tested, which were combinations of three temperature values (40, 60, and 80 °C) and three pressure values (10, 20, and 40 MPa). Portions of the plant material were placed inside a 500-mL vessel and, after 15 min of thermostating, the vessel was filled with carbon dioxide under pre-established parameters. The vessel was left for 2 h to achieve the maximum possible dissolution of furocoumarins in the supercritical fluid. Subsequently, a flow was started, and 25 mL of the carbon dioxide was collected. It was assumed that the concentration of furocoumarins in the collected fluid would be equal to the solubility of the analytes.

The impact of supercritical carbon dioxide parameters on the solubility of furocoumarins was described using the equation presented by Chrastil (Equation (1)) [33]:

(1) $$c=\rho_{f}{}^{k}\times exp\left(\frac{a}{T}+b\right)$$

The equation connects the solubility of the analyte (*c*) with the temperature (*T*) and the density (*ρ_f_*) of the supercritical fluid. Additional parameters of the equation (*a*, *b*, and *k*) relate to the thermodynamic properties of the solvation process. The densities of carbon dioxide under various conditions were calculated using an online tool based on the equation of state presented by Span and Wagner [37]. The solubility values were also recalculated to mass fractions of the analytes in carbon dioxide using Equation (2):

(2) $$y^{*}=c/\rho_{f}$$

### 3.4. Quantification of Furocoumarins

The furocoumarin content was determined using the HPLC method presented by Frérot and Decorzant [38]. Separation of 25 μL samples was performed on a Sunfire C18, 5 μm, 4.6 mm × 250 mm column (Waters), which was maintained at 25 °C. The column was eluted at a flow rate of 0.8 mL min^−1^ using the gradient presented in Table 6. During the analysis, two detectors were used simultaneously: the spectrophotometer (working at wavelengths of 245 nm and 310 nm), and the fluorimeter (excitation wavelength of 310 nm and emission wavelength range of 440–540 nm). The analytes were identified by comparison of retention times with the authentic standards, while quantitative determination of their content was performed using calibration curves at a wavelength of 310 nm. Observed retention times were similar to reported by authors of the method. Unidentified compounds were considered as furocoumarins if strong fluorescence was observed in the analyzed wavelength range.

### 3.5. Mathematical Analysis of Data

The broken plus intact cell (BIC) model presented by Sovová is the most frequently used method of description of the kinetics of supercritical fluid extraction of a plant material [39]. The model assumes that the mechanical pretreatment of the samples results in the presence of two types of cells in the matrix varying in extraction rates. Extraction curves are divided into three subsequent parts based on the kinetic phenomena occurring:

• Constant extraction rate (CER) stage (Equation (3)):

(3) $$x\left(t\right)=y^{*}\times q_{m}\times t\times \left[1-exp\left(-Z\right)\right]$$

• Falling extraction rate (FER) stage (Equation (4)):

(4) $$x\left(t\right)=y^{*}\times q_{m}\times \left[t-t_{CER}\times exp\left(Z_{w}-Z\right)\right]$$

• Diffusion-controlled (DC) stage (Equation (5)):

(5) $$x\left(t\right)=x_{0}-\frac{y^{*}}{W}\times ln\left\{1+\left[exp\left(\frac{W\times x_{0}}{y^{*}}\right)-1\right]\times exp\left[W\times q_{m}\times \left(t_{CER}-t\right)\times r\right]\right\}$$

A detailed description of the model, including its advantages and limitations, can be found in the original paper by Sovová [39] as well as in the subsequent review articles [35]. Three dimensionless parameters and endpoints of the process phases are introduced to simplify the equations of the extraction curves (Equations (6)–(10)). Table 7 presents a description and the method of determination for all the parameters used.

(6) $$Z=\frac{k_{f}\times a_{0}\times \rho_{f}}{q_{m}\times \left(1-\varepsilon \right)\times \rho_{s}}$$

(7) $$W=\frac{k_{s}\times a_{0}}{q_{m}\times \left(1-\varepsilon \right)}$$

(8) $$Z_{w}=\frac{Z\times y^{*}}{W\times x_{0}}\times ln\left[\frac{exp\left[W\times q_{m}\times \left(t-t_{CER}\right)\right]-r}{1-r}\right]$$

(9) $$t_{CER}=\frac{x_{0}\times \left(1-r\right)}{y^{*}\times Z\times q_{m}}$$

(10) $$t_{FER}=t_{CER}+\frac{1}{W\times q_{m}}\times ln\left[r+\left(1-r\right)\times exp\left(\frac{W\times x_{0}}{y^{*}}\right)\right]$$

## 4. Conclusions

The four selected plants proved to be rich sources of furocoumarins. The matrices varied in quantitative and qualitative furocoumarin patterns; the total content of these compounds was in a range of 4.03–26.45 mg per g of dry weight. A total of eight individual compounds was identified, however the analyses showed the presence of other minor compounds in the matrices. Solubility of furocoumarins was investigated in nine sets of carbon dioxide parameters. The solubility grew with an increase of temperature and pressure, which is consistent with literature findings; while the Chrastil’s equation proved suitable to describe the impact of the parameters on the solubility of the furocoumarins. The research presented proves the possibility of extracting furocoumarins from plant sources using supercritical carbon dioxide, while the BIC model was an appropriate method for describing the extraction kinetics. The extraction rate of the furocoumarins was affected by their structure and chemical properties; however, in the majority of the cases, differences between the analytes were negligible from a practical point-of-view. The shape of the extraction curves was affected by solubility of the analytes and mass transfer phenomena. The comparison of curves obtained during extraction of *Psoralea corylifolia* at various process parameters showed that solubility is the most important factor in selection as it impacts the first, most rapid phase of the extraction. Differences in the rate of mass transfer phenomena, which affect the latter part of extraction, have only a limited impact on the process curves.

The work presented could be a starting point for more sophisticated work on the extraction of furocoumarins from plant matrices. The prospective industrial application of this process requires a scaling-up study and an economic comparison with alternative processing methods. Additionally, the selectivity of the method needs to be taken into consideration, as it impacts the further steps of furocoumarin isolation and purification.

## Figures and Tables

**Table 1:** Furocoumarin content in investigated matrices.

	Content (mg g^−1^ (d.w.))			
Compound	*A. archangelica*	*C. paradisi*	*C. monnieri*	*P. corylifolia*
psoralen	nd	nd	nd	13.22
isopsoralen	nd	nd	nd	11.05
bergapten	0.64	0.14	nd	nd
methoxsalen	2.11	nd	4.12	nd
bergamottin	nd	1.63	nd	nd
DHB	nd	2.20	nd	nd
imperatorin	5.85	nd	0.81	nd
osthole	0.43	nd	9.43	nd
other	0.92	0.06	0.22	2.18
total	9.95	4.03	14.58	26.45

nd—not detected; DHB—6′,7′-dihydroxybergamottin; other—unidentified furocoumarins (total); d.w.—dry weight.

**Table 2:** Solubility of furocoumarins in supercritical carbon dioxide at a temperature of 80 °C and pressure of 40 MPa.

Plant	Compound	c (g L^−1^)	y* (-)
*A. archangelica*	imperatorin	0.334	4.056 × 10^−4^
	methoxsalen	0.183	2.222 × 10^−4^
	bergapten	0.168	2.040 × 10^−4^
	total	0.685	8.318 × 10^−4^
*C. paradisi*	DHB	0.072	0.874 × 10^−4^
	bergamottin	0.467	5.665 × 10^−4^
	total	0.539	6.539 × 10^−4^
*C. monnieri*	osthole	0.265	3.218 × 10^−4^
	methoxsalen	0.176	2.137 × 10^−4^
	imperatorin	0.341	4.141 × 10^−4^
	total	0.782	9.496 × 10^−4^
*P. corylifolia*	psoralen	0.248	3.011 × 10^−4^
	isopsoralen	0.233	2.829 × 10^−4^
	total	0.481	5.840 × 10^−4^

DHB—6′,7′-dihydroxybergamottin.

**Table 3:** Kinetic parameters for the extraction of furocoumarins with supercritical carbon dioxide at a temperature of 80 °C and pressure of 40 MPa.

Plant	Compound	r (-)	k_s_ (m s^−1^)	k_f_ (m s^−1^)
*A. archangelica*	imperatorin	0.244	2.33 × 10^−8^	2.43 × 10^−6^
	methoxsalen		2.84 × 10^−8^	2.71 × 10^−6^
	bergapten		2.39 × 10^−8^	2.44 × 10^−6^
*C. paradisi*	DHB	0.303	1.22 × 10^−8^	1.81 × 10^−6^
	bergamottin		1.14 × 10^−8^	1.95 × 10^−6^
*C. monnieri*	osthole	0.307	2.95 × 10^−8^	2.38 × 10^−6^
	methoxsalen		2.83 × 10^−8^	2.73 × 10^−6^
	imperatorin		2.41 × 10^−8^	2.53 × 10^−6^
*P. corylifolia*	psoralen	0.225	3.06 × 10^−8^	4.12 × 10^−6^
	isopsoralen		3.01 × 10^−8^	4.27 × 10^−6^

DHB—6′,7′-dihydroxybergamottin.

**Table 4:** Kinetic parameters for the isolation of furocoumarins from *P. corylifolia* with using a temperature of 80 °C and pressure of 40 MPa.

Parameters	Compound	y* (-)	k_s_ (m s^−1^)	k_f_ (m s^−1^)
60 °C, 20 MPa	psoralen	0.794 × 10^−4^	2.88 × 10^−8^	3.95 × 10^−6^
	isopsoralen	0.742 × 10^−4^	2.91 × 10^−8^	3.92 × 10^−6^
60 °C, 40 MPa	psoralen	1.115 × 10^−4^	2.46 × 10^−8^	3.62 × 10^−6^
	isopsoralen	1.056 × 10^−4^	2.44 × 10^−8^	3.61 × 10^−6^
80 °C, 20 MPa	psoralen	1.767 × 10^−4^	3.21 × 10^−8^	4.68 × 10^−6^
	isopsoralen	1.649 × 10^−4^	3.24 × 10^−8^	4.71 × 10^−6^
80 °C, 40 MPa	psoralen	3.011 × 10^−4^	3.06 × 10^−8^	4.12 × 10^−6^
	isopsoralen	2.829 × 10^−4^	3.01 × 10^−8^	4.27 × 10^−6^

**Table 5:** Plant material used during the research.

Scientific Name	Common Name	Material	Country of Origin	Moisture Content
*Angelica archangelica* L.	garden angelica	dried root	Poland	8.2%
*Citrus × paradisi Macfad.*	grapefruit	dried pomace	Italy	8.8%
*Cnidium monnieri* L.	she chuangzi	seeds	China	5.7%
*Psoralea corylifolia* L.	babchi	seeds	India	4.7%

**Table 6:** Gradient used during HPLC analysis of the furocoumarins.

Time (min)	Eluent A Water-Acetonitrile-THF 85:10:5 (vol%)	Eluent B Acetonitrile-Methanol-THF 65:30:5 (vol%)
0.0	100%	0%
7.5	100%	0%
30.0	68%	32%
36.0	68%	32%
57.0	45%	55%
60.0	10%	90%
75.0	10%	90%
90.0	100%	0%
95.0	100%	0%

**Table 7:** Parameters used during the research.

Parameter	Description	Method of Determination
*values selected by experimenters*		
T (°C)	temperature of process	-
P (MPa)	pressure of process	-
q_v_ (L s^−1^)	volumetric flow of CO_2_	-
t (s)	time of extraction	-
m_s_ (g)	mass of sample	-
V_b_ (L)	volume of vessel	-
*characteristics of extraction bed*		
d (m)	average diameter of particle	microscopically (Section 3.2)
ρ_s_ (g L^−1^)	density of sample	pycnometer
ρ_a_ (g L^−1^)	apparent density of bed	m_s_/V_b_
ε (-)	porosity of bed	1-(ρ_a_/ρ_s_)
a_0_ (m^−1^)	specific surface area	6(1-ε)/d
*characteristics of supercritical fluid*		
ρ_f_ (g L^−1^)	density of CO_2_	calculated [16]
q_m_ (s^−1^)	relative mass flow of CO_2_	(q_v_* ρ_f_)/ m_s_
y* (-)	solubility of analyte	experimentally (Section 3.3.3)
*characteristics of extracts*		
x_0_ (-)	relative total content of analyte	HPLC of Soxhlet extracts
x (-)	relative yield of extraction	HPLC of SFE extracts
*main kinetic parameters of extraction*		
r (-)	fraction of less accessible solute	regressed from experimental data
k_f_ (m s^−1^)	solvent-phase mass transfer coefficient	
ks (m s^−1^)	solid-phase mass transfer coefficient	
*auxiliary parameters*		
W (-)	dimensionless parameter	Equation (6)
Z (-)	dimensionless parameter	Equation (7)
Z_w_ (-)	dimensionless parameter	Equation (8)
t_CER_ (s)	endpoint of CER stage	Equation (9)
t_FER_ (s)	endpoint of FER stage	Equation (10)

## Supplementary Materials

The Supplementary Materials are available online.
