# Supplementary material for: Photosensitizing Furocoumarins: Content in Plant Matrices and Kinetics of Supercritical Carbon Dioxide Extraction
Source: Molecules. 2020 Aug 21;25(17):3805. doi: 10.3390/molecules25173805 (PMC7503550; doi:10.3390/molecules25173805)
Supplement: Supplementary file 1 [file molecules-25-03805-s001.pdf]

Table S1. Characteristics of the extraction beds. Symbols as presented in Table 7.

Plant	d (m)	ϱ_s (g L^{-1})	ε (-)	a_0 (m^{-1})
A. archangelica	7.2 10^{-4}	680	0.461	4490
C. paradisi	1.9 10^{-3}	616	0.395	1910
C. monnieri	3.9 10^{-4}	721	0.294	10860
P. corylifolia	2.3 10^{-4}	703	0.405	15520

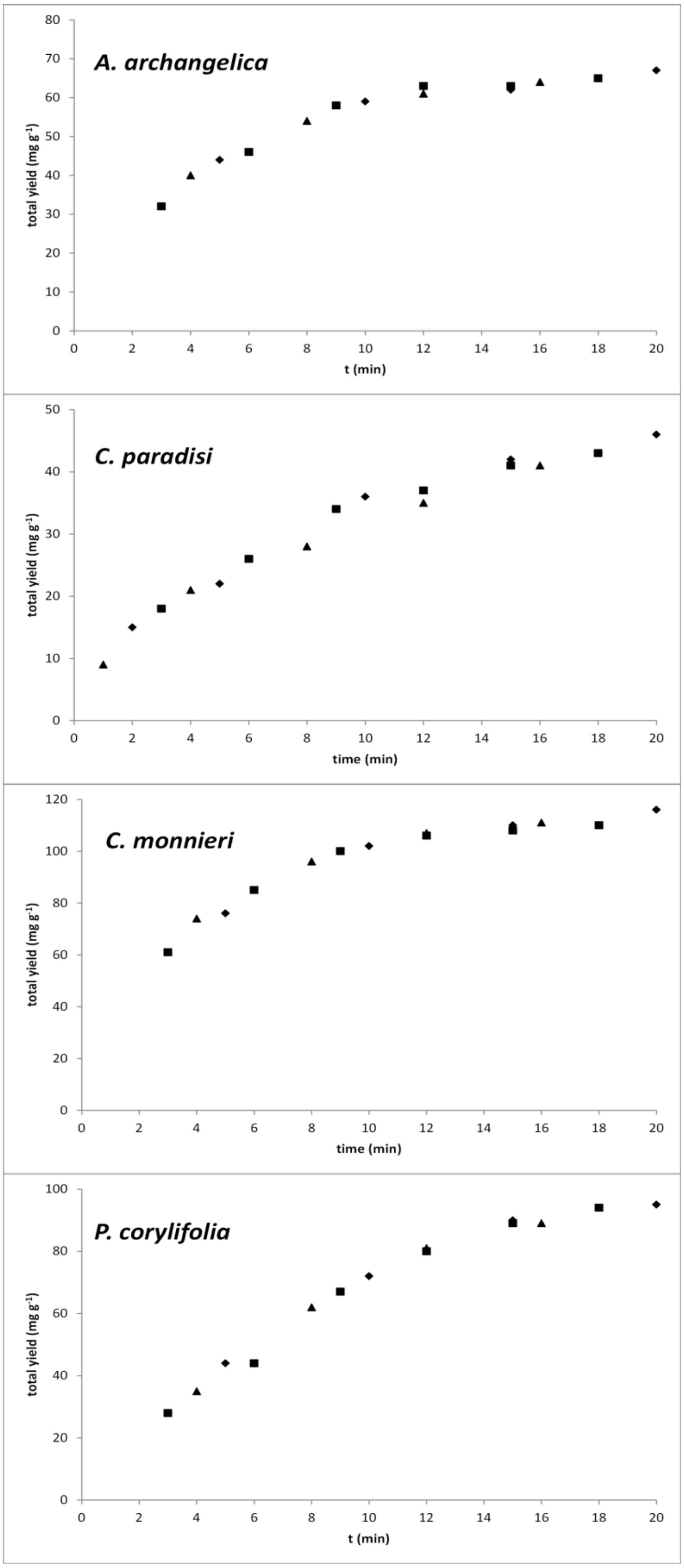

Figure S1. Total extraction curves for plant tissues at a temperature of 80 °C and pressure of 40 MPa. The black points (squares, triangles, and diamonds) represent experimental data from three processes.
